# The Drug Treatment of Insomnia

**Published:** 1898-02

**Authors:** 


### THE DRUG TREATMENT OF INSOMNIA.

Dr. R. Ferguson, lecturer on therapeutics in the Western University (*British Medical Journal*), presented at the last meeting of the British Medical Association some practical suggestions on the use of drugs in the treatment of insomnia. While he did not believe that the hypnotic is yet discovered, or ever will be, which is at once trustworthy as to producing the result desired and incapable of producing any unpleasant after-effects, sulfonal, in his opinion, came as near to this standard as any drug with which he was acquainted. The tardiness and permanency of its action was undoubtedly a consequence of its difficult solubility and slow absorption, and could not be looked upon as a disadvantage if properly allowed for, since a second good night without a repetition of the dose was by no means an unusual occurrence. In the author's practice it had failed but rarely when given in the dose of 1 gramme. He had rarely, if ever, repeated this dose on the same night. In doses of less than thirty centigrammes it might be properly considered a semi-placebo. It should not be given continuously, that is, on successive nights, for any length of time and if its use has to be continued the intervals should be even longer than two days. The peculiarities of action of sulfonal could be best utilized by reserving it for a patient who had not slept for several nights and who it was pretty clear was not going to get a good night unassisted, so that the medicine may be given early—namely, 6 to 7 P. M., in order that its effect may be developed at about the regular time for going to sleep—*Buffalo Medical Journal.*

